# The state of the HIV epidemic in rural KwaZulu-Natal, South Africa: a novel application of disease metrics to assess trajectories and highlight areas for intervention

**DOI:** 10.1093/ije/dyz269

**Published:** 2020-01-13

**Authors:** Alain Vandormael, Diego Cuadros, Hae-Young Kim, Till Bärnighausen, Frank Tanser

**Affiliations:** 1 Africa Health Research Institute (AHRI), KwaZulu-Natal, Durban, South Africa; 2 School of Nursing and Public Health, University of KwaZulu-Natal (UKZN), Durban, South Africa; 3 KwaZulu-Natal Research Innovation and Sequencing Platform (KRISP), UKZN, Durban, South Africa; 4 Heidelberg Institute of Global Health, Faculty of Medicine, University of Heidelberg, Heidelberg, Germany; 5 Department of Geography and Geographic Information Science, University of Cincinnati, Cincinnati, OH, USA; 6 Department of Population Health, New York University School of Medicine, New York, USA; 7 Department of Global Health and Population, Harvard T.H. Chan School of Public Health, Boston, MA, USA; 8 Lincoln Institute for Health, University of Lincoln, Lincoln, UK; 9 Centre for the AIDS Programme of Research in South Africa (CAPRISA), UKZN, Durban, South Africa

**Keywords:** HIV, epidemic control, incidence-mortality ratio, incidence-prevalence ratio, UNAIDS, South Africa

## Abstract

**Background:**

South Africa is at the epicentre of the HIV pandemic, with the world's highest number of new infections and the largest treatment programme. Using metrics proposed by the Joint United Nations Programme on AIDS (UNAIDS), we evaluate progress toward epidemic control and highlight areas for intervention in a hyperendemic South African setting.

**Methods:**

The Africa Health Research Institute (AHRI) maintains a comprehensive population-based surveillance system in the Hlabisa sub-district of KwaZulu-Natal. Between 2005 and 2017, we tested 39 735 participants (aged 15–49 years) for HIV and followed 22 758 HIV-negative and 13 460 HIV-positive participants to identify new infections and all-cause AIDS-related deaths, respectively. Using these data, we estimated the percentage reduction in incidence, the absolute incidence rate, the incidence-mortality ratio and the incidence-prevalence ratio over place and time.

**Results:**

We observed a 62% reduction in the number of new infections among men between 2012 and 2017 and a 34% reduction among women between 2014 and 2017. Among men, the incidence-mortality ratio peaked at 4.1 in 2013 and declined to 3.1 in 2017, and among women it fell from a high of 6.4 in 2014 to 4.3 in 2017. Between 2012 and 2017, the female-incidence/male-prevalence ratio declined from 0.24 to 0.13 and the male-incidence/female-prevalence ratio from 0.05 to 0.02.

**Conclusions:**

Using data from a population-based cohort study, we report impressive progress toward HIV epidemic control in a severely affected South African setting. However, overall progress is off track for 2020 targets set by the UNAIDS. Spatial estimates of the metrics, which demonstrate remarkable heterogeneity over place and time, indicate areas that could benefit from additional or optimized HIV prevention services.

**Key Messages**

- Using metrics proposed by the Joint United Nations Programme on AIDS (UNAIDS), we evaluate progress toward HIV epidemic control in a severely affected South African setting. Between 2005 and 2017, we tested 39 735 participants (aged 15–49 years) for HIV and followed 22 758 HIV-negative and 13 460 HIV-positive participants from a large population-based cohort in rural KwaZulu-Natal.
- For the first time, we use data from a population-based cohort to estimate the percentage reduction in incidence, the absolute incidence rate, the incidence-mortality ratio and the incidence-prevalence ratio. Our results, which are derived from empirical data rather than mathematical models, show remarkable heterogeneity in the four metrics over place and time.
- There has been impressive progress toward HIV epidemic control in this hyperendemic South African setting. Nevertheless, expanded and sustained prevention services are needed to reach key UNAIDS milestones. Our results highlight geographical areas that could benefit from these prevention services.

## Introduction

In 2014, the Joint United Nations Programme on AIDS (UNAIDS) announced a set of ambitious but achievable targets to end the HIV epidemic by 2030.^1^ To assess interim progress, a scientific panel recently convened in Glion, Switzerland, to build consensus around the measurement of epidemic control, defined as a reduction of disease incidence, prevalence or mortality in a geographical area to a locally acceptable level.^2^^,^^3^ The panel proposed four metrics called the percentage reduction in incidence, the absolute incidence rate, the incidence-prevalence ratio and the incidence-mortality ratio, each having a target to galvanize political support and promote real-world impact in the fight against HIV.

Progress toward HIV epidemic control has generally been measured through reductions in the number of new infections and/or declines in the absolute incidence rate.^4–9^ The incidence-prevalence ratio, defined as the number of new infections occurring over the lifetime of an infected person on treatment, has also been used, but less frequently and in high-income countries.^10–12^ Empirical estimates are currently unavailable for the incidence-mortality ratio, which measures the number of new infections relative to the number of all-cause HIV-related deaths.^13^ Using a modelling approach, the UNAIDS recently estimated the four metrics^14^ and Galvani *et al*.^15^ assessed their strengths and weaknesses; Ghys *et al.*^16^ also provided a conceptual overview. To date, however, the four metrics have not been estimated from empirical data to evaluate the state of the HIV epidemic at the population level.

In this study, we present temporal and geospatial estimates of the four epidemic metrics from South Africa, the country most affected by the HIV pandemic.^4^ South Africa is of considerable interest, having received substantial domestic and foreign investment to scale up the world’s largest HIV treatment programme.^4^^,^^17^ To evaluate progress toward epidemic control, we tested 39 735 participants (aged 15–49 years) for HIV, and followed a cohort of 22 758 HIV-negative and 13 460 HIV-positive participants from a hyperendemic setting in rural KwaZulu-Natal.

## Methods

### Data collection

The Africa Health Research Institute (AHRI) maintains a comprehensive demographic surveillance system in the sub-district of Hlabisa. The surveillance area is 438 km^2^ in size and home to approximately 90 000 residents and 11 000 households. Households are mostly scattered across the predominantly rural landscape, with several peri-urban settlements and a single urban township.

Twice yearly, trained fieldworkers visit all households in the surveillance area to interview a key informant. The household head or the most senior household member is often the key informant; if not available, other suitable household members are selected. The key informant provides information on: the physical attributes of the household; the resident members and their relationship to one another; members who join, leave or die; and the migration patterns of members, including place of origin and destination. For each death in the household, the key informant completes a detailed verbal autopsy questionnaire administered by the fieldworker. Household response rates are typically *>*95%.^18^

Nested within the household surveillance survey is an annual HIV survey that has been ongoing since 2004. All persons who reside in the surveillance area and are older than 15 years are eligible for HIV testing. After obtaining written consent, the fieldworkers interview participants and extract blood according to the *UNAIDS and WHO Guidelines for Using HIV Testing Technologies in Surveillance*.^19^ The dried blood spots are transported to the AHRI laboratory in Durban where HIV status is determined by antibody testing with a broad-based HIV-1/HIV-2 enzyme-linked immunosorbent assay (ELISA) (Vironostika HIV-1 Microelisa System: Biomérieux, Durham, NC, USA) followed by a second ELISA (Wellcozyme HIV1 + 2 GACELISA: Murex Diagnostics Benelux B.V., Breukelen, The Netherlands).

Antiretroviral therapy (ART) services have been freely available at 17 public health care clinics within or adjacent to the surveillance area since 2004, with a CD4+ T cell count eligibility criterion of *<*200 cells/µL. All patients with CD4+ T cell counts *<*350 cells/µL became eligible for ART in 2011. In 2015, ART was made available to HIV-positive pregnant women regardless of CD4+ T cell count, and to all late adolescents and adults with CD4+ T cell counts *<*500 cells/µL. CD4+ eligibility criteria were removed in September 2016.^20^ AHRI has linked ART usage data collected at the health care clinics to the surveillance database.

### Metrics

We calculated the percentage reduction in new infections, the absolute incidence rate, the incidence-mortality ratio and the incidence-prevalence ratio for men and women aged 15–49 years. Detailed notation is provided in the Methods section of the Supplement, available as Supplementary data at *IJE* online. We define $N_{y}^{T}$ as the total number of participants (irrespective of HIV testing status) who were residents in the surveillance area for >50% of the *y*th year (*y *=* *2005, … *,* 2017). Since all measures are calculated by year, we drop the *y* subscript for convenience. Let *N* denote the number of participants who tested for HIV. From the *N* participants, we calculated the HIV-positive prevalence (*H*^+^) and the HIV-negative prevalence (*H^−^*) as proportions. Further, let *E*^+^ denote the expected number of HIV-positive participants, where $E^{+}=N^{T}\times H^{+},$ and let *E^−^* denote the expected number of HIV-negative participants, where $E^{-}=N^{T}\times H^{-}.\;$ We use these measures to derive the four epidemic control metrics.

To calculate the absolute incidence rate, we identified all participants with a first HIV-negative result followed by at least one valid HIV test result during the observation period. We recorded the exposure time and the number of repeat-testers who converted from an HIV-negative to an HIV-positive result. We then calculated the incidence rate per 100 person-years, denoted by *IR*, using methods described elsewhere.^21^ A well-cited target for epidemic control is to decrease the absolute incidence rate to less than one new HIV event per 1000 uninfected adults or person-years.^22^^,^^23^ To calculate the expected number of new infections, we multiplied the absolute incidence rate by the expected number of HIV-negative participants: *I* = *IR × E^−^*. We used this result to obtain the percentage change in the expected number of new infections over a given time period, defined as $I\%=\left(I_{y2}-I_{y1}\right)/I_{y1}\times 100$, where the subscripts $y_{1}$ and $y_{2}$ denote an earlier and later year, respectively. Targets for percentage reductions will vary by country and scale of the local epidemic. The UNAIDS, for example, aims to reduce the global number of new HIV infections by 75% between 2010 and 2020.^1^

To obtain the incidence-mortality ratio, we followed all HIV-positive participants and recorded the survival time and number of all-cause related deaths. We denote the HIV mortality rate by *M.* Next, we calculated the expected number of deaths, *D*, by multiplying the HIV-related mortality rate by the expected number of HIV-positive participants: $D=M\times E^{+}.$ The incidence-mortality ratio is given as *IMR* = I/D with an epidemic control threshold <1, which is achieved when the number of new HIV infections (numerator) falls below the number of all-cause HIV-related deaths (denominator) in a given year.^13^

For the incidence-prevalence ratio, we divided the expected number of new HIV-infected participants by the expected number of opposite-sex HIV-positive participants, such that the *IPR* = $I/E^{+}$. The *IPR* threshold for epidemic control is <0.03, which assumes that the average survival time of a newly infected person on ART is 33 years. To achieve epidemic control, fewer than one new infection should occur over the 33-year-period, which translates into 1/33 or three new infections per 100 people living with HIV per year.^2^^,^^16^ Because of the generalized, heterosexual epidemic in sub-Saharan Africa, we used opposite-sex versions of the incidence-prevalence ratio, since new male infections are largely related to the number of HIV-positive females and vice versa.

Using the same methodology as above, we computed geospatial versions of the four epidemic control metrics. To do this, we used a moving two-dimensional Gaussian kernel of 3-km search radius,^24^ the size of which was determined from previous work.^25^ We identified the household coordinates of all participants and superimposed the expected number of new HIV infections and the expected number of AIDS-related deaths on a geographical representation of the study area consisting of a grid of 1 km x 1 km pixels. For each year, we calculated Gaussian weighted estimates of the above measures and generated a raster grid for each. Next, we calculated *I* by multiplying the raster grids of *IR*, *H^−^* and *N^T^*. We calculated the incidence-mortality ratio by dividing the raster grid of *I* by the raster grid of *D*, and used a similar procedure for the opposite-sex incidence-prevalence ratio. We undertook all statistical computations using R version 3.6.2, and all raster grid computations using map algebra in ArcGIS version 10.5.^26^

### Ethical approval

Ethics approval for data collection and use was obtained from the biomedical and ethics committee (BREC) of the University of KwaZulu-Natal (Durban, South Africa), BREC approval number BE290/16.

## Results

Table 1 shows the number of participants who tested in the HIV survey by year. A detailed description of the HIV participation rates is provided elsewhere.^6^ The mean age of the HIV testers was 27 [interquartile range (IQR): 18–35] years and approximately 60% of these participants were women. Between 2005 and 2017, the percentage of participants who tested at least once for HIV increased from 42% to 79%. During this period, ART coverage increased from 2% to 45%.

**Table 1.** Particiation rates for all persons (aged 15–49 years) in the HIV surveillance survey, the HIV incidence cohort and the HIV mortality cohort

	HIV survey^a^			HIV incidence cohort^b^		HIV mortality cohort^c^	
	Tested	Ever	ART coverage	HIV^–^	Entered	HIV^+^	Entered
Year	N (% Female)	Tested	%	Eligible	N (%)	Eligible	N (%)
2005	9363 (60.1)	42.5	2.0	13 212	9697 (73.4)	3940	3704 (94.0)
2006	8212 (61.3)	51.5	5.1	15 453	11 240 (72.7)	4696	4194 (89.3)
2007	7161 (63.4)	53.8	9.8	17 271	12 514 (72.5)	5441	4661 (85.7)
2008	7203 (62.7)	56.4	14.2	19 009	13 732 (72.2)	6167	5044 (81.8)
2009	6215 (64.4)	61.9	18.7	20 003	14 352 (71.8)	6818	5377 (78.9)
2010	7978 (65.3)	64.7	23.4	22 276	15 842 (71.1)	7829	6021 (76.9)
2011	7219 (64.3)	67.8	28.7	23 747	16 684 (70.3)	8564	6403 (74.8)
2012	5430 (64.5)	67.2	33.7	25 172	17 616 (70.0)	9137	6631 (72.6)
2013	6902 (64.9)	70.4	37.4	27 101	18 877 (69.7)	9934	7028 (70.7)
2014	6473 (66.0)	72.2	40.6	28 578	19 912 (69.7)	10 670	7382 (69.2)
2015	9115 (66.4)	76.3	45.3	30 621	21 185 (69.2)	11 665	7993 (68.5)
2016	10 348 (67.7)	79.0	46.0	32 880	22 271 (67.7)	12 794	8665 (67.7)
2017	7609 (66.3)	79.3	44.9	34 232	22 758 (66.5)	13 460	8736 (64.9)

a Shows the number of participants who tested for HIV and the percentage who were women. Ever tested represents the percentage of participants who tested at least once for HIV. ART coverage is the percentage of HIV-positive participants on treatment.

b Shows the number and percentage of eligible HIV-negative participants who entered into the HIV incidence cohort. Participants must have had a first HIV-negative test follwed by at least one HIV test result.

c Shows the number and percentage of eligible HIV-positive participants who entered into the HIV mortality cohort.

Of the eligible HIV-negative participants, 71% (minimum–maximum range: 66–73%) entered the HIV incidence cohort and contributed person-time to the analysis (Table 1). Among the 22 758 participants in the HIV-negative cohort, we observed 3420 seroconversions over 96 303 person-years of follow-up, with an overall incidence rate of 3.53 seroconversions per 100 person-years. Of the eligible HIV-positive participants, 77% (minimum–maximum range: 65–94%) entered the HIV mortality cohort and contributed person-time to the analysis. For the HIV mortality cohort, we observed 1672 deaths over 74 749 person-years of follow-up, with a mortality rate of 2.24 deaths per 100 person-years.

**Figure 1.**
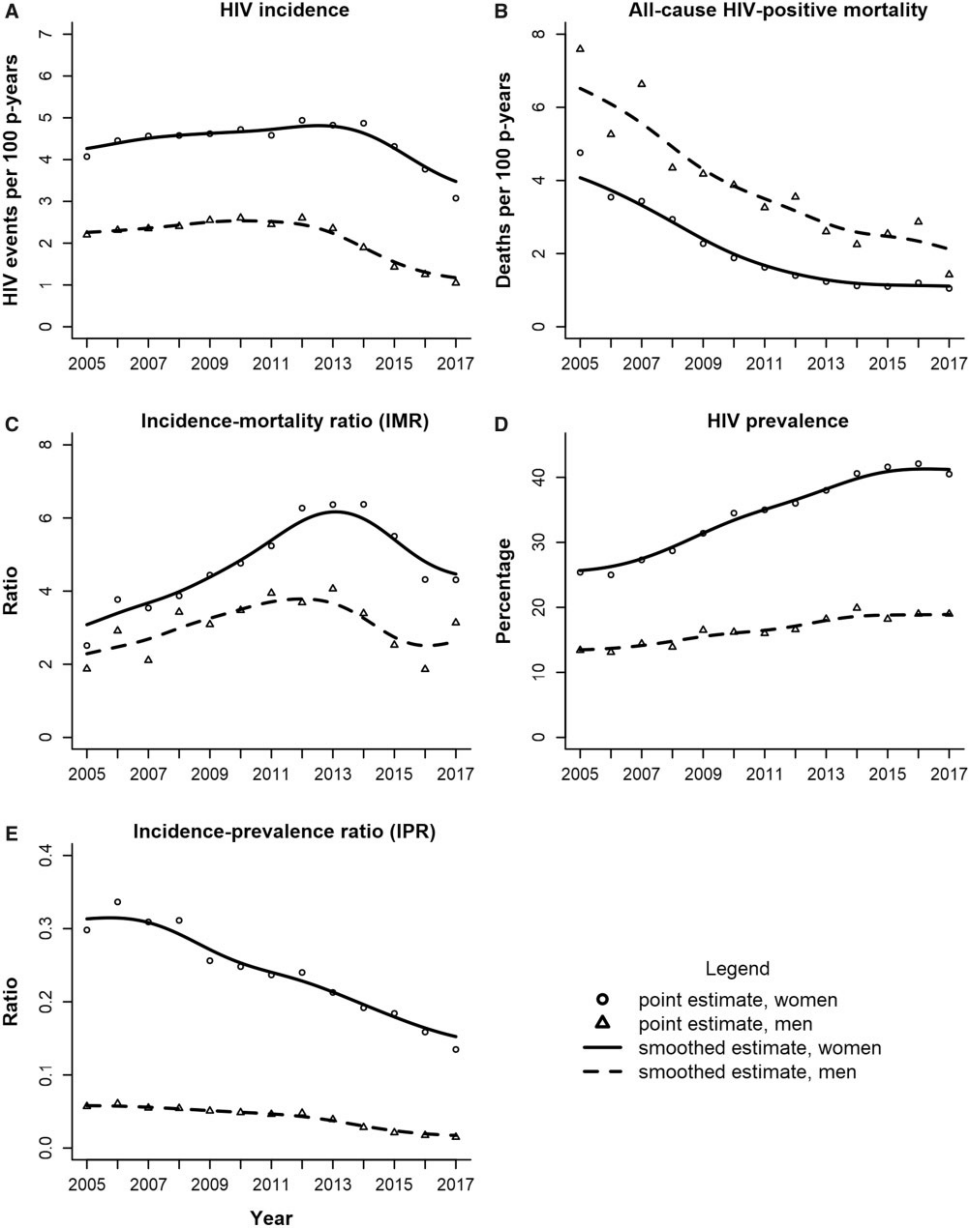
Temporal trends in the HIV incidence rate (Panel A), all-cause HIV-positive mortality rate (Panel B), incidence-mortality ratio (Panel C) and HIV prevalence (Panel D) by sex in the Africa Health Research Institute surveillance area (2005–17). Panel E shows the (opposite-sex) female-incidence/male-prevalence (circle and solid line) and the male-incidence/female-prevalence (triangle and dashed line) ratios.

The summary statistics used to calculate the epidemic metrics for all participants are shown in Supplementary Table S1 and Supplementary Figure S1, available as Supplementary data at *IJE* online. Table 2 shows these results by sex. The first column represents the number of men and women aged 15–49 years ($N^{T}$) who resided in the surveillance area. Also shown are the HIV-positive and HIV-negative prevalences (numerators and denominators not shown) and the expected number of HIV-negative and HIV-positive participants. Among men, the absolute HIV incidence rate declined from 2.6 to 1.1 events per 100 person-years between 2012 and 2017. HIV incidence declines occurred 2 year later among women, falling from 4.9 to 3.1 events per 100 person-years between 2014 and 2017. Trends in the absolute HIV incidence rate by men and women are shown in Panel A of Figure 1. Among men, there was a 62% reduction in the expected number of new HIV infections between 2012 ($I_{1}=\;$657 infections) and 2017 ($I_{2}=$251 infections). Among women, the expected number of new HIV infections increased by 8% between 2005 and 2013, before falling by 34% between 2014 ($I_{1}=\;$1146 infections) and 2017 ($I_{2}=\;$758 infections).

**Table 2.** Summary of the epidemic metrics by men and women in the Africa Health Research Institute surveillance area, 2005–17

	HIV survey^a^					Incidence cohort^b^			Mortality cohort^c^				
Year	*N* ^T^	HIV^–^	HIV^+^	Expected	Expected	HIV Inf.	HIV Inc.	Expected	Deaths/	Mortality	Expected		
		Prev. %	Prev. %	HIV^–^*N*	HIV^+^*N*	Person-years	Rate	HIV Inf. *N*	Person-years	Rate	Deaths *N*	IMR^d^	IPR^e^
**Men**													
2005	27 551	86.6	13.4	23 859	3691	67/3047	2.20	525	60/791	7.59	280	1.87	0.057
2006	27 280	86.9	13.1	23 706	3573	78/3347	2.31	548	51/969	5.26	188	2.91	0.061
2007	26 972	85.6	14.4	23 088	3883	81/3412	2.35	543	69/1041	6.63	257	2.11	0.055
2008	27 769	86.1	13.9	23 909	3859	83/3445	2.40	575	50/1151	4.35	168	3.43	0.054
2009	28 461	83.5	16.5	23 764	4696	83/3259	2.55	606	52/1245	4.18	196	3.09	0.051
2010	30 750	83.8	16.2	25 768	4981	83/3167	2.60	671	51/1316	3.88	193	3.48	0.049
2011	30 942	84.0	16.0	25 991	4950	75/3029	2.45	637	48/1473	3.26	161	3.95	0.046
2012	30 234	83.4	16.6	25 215	5018	75/2848	2.60	657	54/1521	3.55	178	3.69	0.048
2013	30 757	81.8	18.2	25 159	5597	68/2862	2.35	592	42/1614	2.60	146	4.06	0.039
2014	30 046	80.1	19.9	24 066	5979	55/2889	1.89	456	38/1689	2.25	134	3.39	0.028
2015	31 605	81.8	18.2	25 852	5752	41/2822	1.43	370	46/1809	2.54	146	2.53	0.021
2016	31 574	81.0	19.0	25 574	5999	31/2474	1.25	320	55/1918	2.87	172	1.86	0.017
2017	29 596	81.0	19.0	23 972	5623	20/1878	1.05	251	28/1965	1.42	80	3.14	0.015
**Women**													
2005	36 248	74.6	25.4	27 041	9206	186/4568	4.07	1101	110/2312	4.76	438	2.51	0.300
2006	36 002	75.0	25.0	27 001	9000	223/5010	4.45	1202	98/2765	3.54	319	3.77	0.340
2007	36 183	72.7	27.3	26 305	9877	232/5079	4.56	1201	105/3056	3.44	339	3.54	0.310
2008	36 814	71.3	28.7	26 248	10 565	231/5050	4.58	1201	101/3438	2.94	310	3.87	0.310
2009	37 962	68.6	31.4	26 041	11 920	223/4818	4.62	1203	84/3695	2.27	271	4.44	0.260
2010	39 978	65.5	34.5	26 185	13 792	221/4685	4.72	1236	75/3987	1.88	259	4.76	0.250
2011	39 364	65.0	35.0	25 586	13 777	210/4569	4.58	1172	73/4494	1.62	224	5.24	0.240
2012	38 111	64.0	36.0	24 391	13 719	214/4321	4.94	1205	65/4642	1.40	192	6.27	0.240
2013	39 820	62.0	38.0	24 688	15 131	211/4358	4.82	1191	61/4934	1.24	187	6.37	0.210
2014	39 646	59.4	40.6	23 549	16 096	211/4333	4.87	1147	58/5188	1.12	180	6.37	0.190
2015	42 038	58.4	41.6	24 550	17 487	183/4232	4.31	1059	61/5541	1.10	193	5.50	0.180
2016	43 605	57.9	42.1	25 247	18 357	143/3786	3.77	951	72/5998	1.20	220	4.32	0.160
2017	41 416	59.5	40.5	24 642	16 773	93/3006	3.08	758	65/6198	1.05	176	4.31	0.130

a $N^{T}$ gives the total number of participants who resided for >50% of the year in the surveillance area (irrespective of consent to HIV testing). HIV^+^ Prev. and HIV^−^ Prev. represent the HIV-positive and HIV-negative prevalence, respectively. The expected number of HIV-negatives (column 5) is obtained by multiplying $N^{T}$(column 2) by the HIV-negative prevalence (column 3). The expected number of HIV-positives (column 6) is obtained by multiplying $N^{T}$ (column 2) by the HIV-positive prevalence (column 4).

b Shows the number of observed HIV infections (HIV Inf.) and person-years of observation (column 7). The HIV incidence (HIV Inc.) rate is per 100 person-years (column 8). The expected number of new HIV infections (column 9) is obtained by multiplying the expected number of HIV-negatives (column 5) by the HIV incidence rate/100 (column 8).

c Shows the number of observed deaths among HIV-positive persons and the person-years of observation (column 10). The HIV-related mortality rate is per 100 person-years (column 11). The expected number of HIV-related deaths (column 12) is obtained by multiplying the expected number of HIV-positives (column 6) by the HIV-mortality rate/100 (column 11).

d The incidence-mortality ratio (IMR, column 13) is obtained by dividing the expected number of new HIV infections (column 9) by the expected number of HIV-related deaths (column 12).

e The incidence-prevalence ratio (IPR, column 14) is obtained by dividing the expected number of new HIV infected participants (column 9, e.g. males) by the expected number of opposite-sex HIV-positive participants (column 6, e.g. females).

The number of HIV-related deaths, the person-years contributed by HIV-positive participants and the expected number of HIV-related deaths are shown in Table 2. Panel B in Figure 1 shows significant reductions in the all-cause mortality rate among HIV-positive men and women. Among HIV-positive women, the all-cause mortality rate dropped from 4.8 to 1.1 deaths per 100 person-years between 2005 and 2017 (‘Mort. Rate’ in Table 2). Nevertheless, the female incidence-mortality ratio was high, rising to 6.4 in 2013 before falling to 4.3 in 2017 (‘IMR’ in Table 2 and Panel C of Figure 1). The incidence-mortality ratio was lower among men, which peaked at 4.1 in 2013 and closed at 3.1 in 2017.

Consistent with a declining all-cause AIDS mortality rate, we observed a steady increase in the prevalence of HIV among men and women (Panel D of Figure 1). Between 2012 and 2017, the female-incidence/male-prevalence ratio declined from 0.24 to 0.13 and the male-incidence/female-prevalence ratio, which was comparatively lower, declined from 0.05 to 0.02 (‘IPR’ in Table 2). These opposite-sex incidence-prevalence ratios confirm the disproportionate burden of HIV among women relative to men when compared with their same-sex versions, as shown in Supplementary Figure S2, available as Supplementary data at *IJE* online. Despite a steady decline in the overall incidence-prevalence ratio, it remained above the epidemic threshold of 0.03, reaching 0.08 in 2017 (Panel E of Supplementary Figure S1 and ‘IMR’ in Supplementary Table S1).

**Figure 2.**
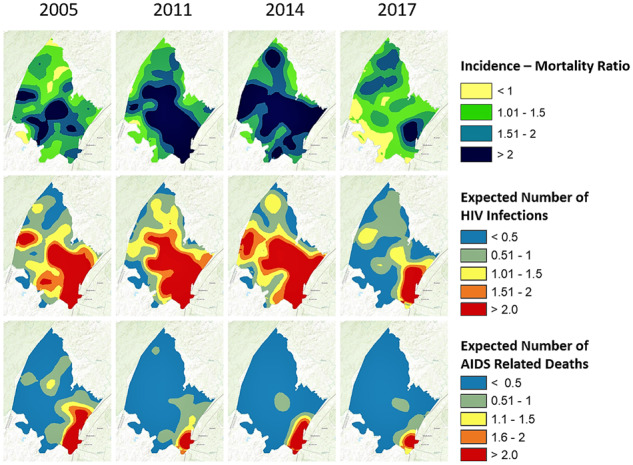
Spatial representation of the incidence-mortality ratio, expected number of new HIV infections (per 1 km by 1 km) and expected number of AIDS-related deaths (per 1 km by 1 km) among women for 2005, 2011, 2014 and 2017.

**Figure 3.**
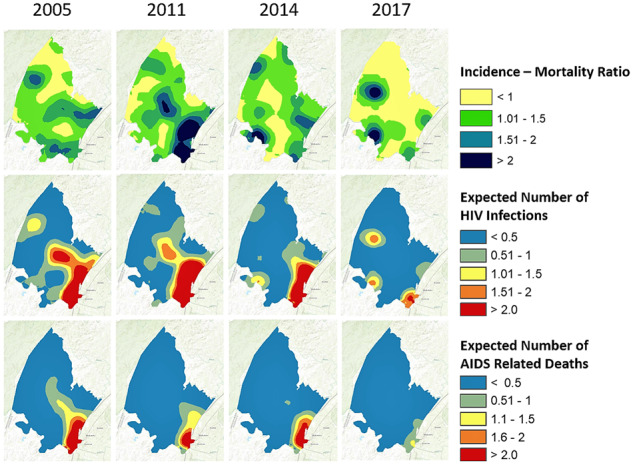
Spatial representation of the incidence-mortality ratio, expected number of new HIV infections (per 1 km by 1 km) and expected number of AIDS-related deaths (per 1 km by 1 km) among men for 2005, 2011, 2014 and 2017.

**Figure 4.**
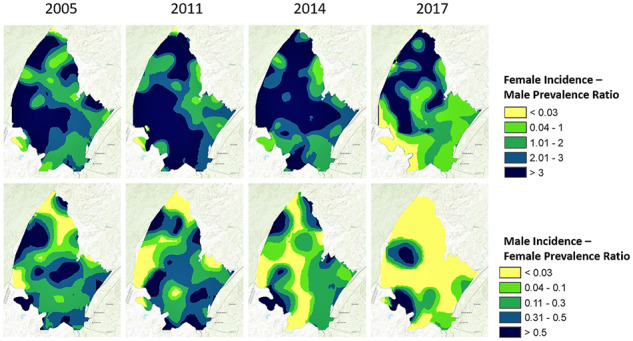
Spatial representation of the female-incidence/male-prevalence ratio (top row) and male-incidence/female-prevalence ratio (bottom row) for 2005, 2011, 2014 and 2017.

Spatial estimates of the incidence rate, the incidence-mortality ratio and the incidence-prevalence ratio varied substantially over time. Among women, less than 5% of the surveillance area had an incidence-mortality ratio <1 in 2014, which increased to 10% in 2017 (Figure 2, Row 1). Among men, an incidence-mortality ratio <1 was prevalent in 30% of the surveillance area in 2014, which increased to 47% in 2017 (Figure 3, Row 1). Increases in areas with an incidence-mortality ratio <1 correspond with large temporal declines in the HIV incidence rate during this period, as shown in Figures 2 and 3 (Row 2). The prevalence of areas with a male-incidence/female-prevalence ratio <0.03 increased from 16% in 2011 to 43% in 2014 and to 62% in 2017. The female-incidence/male-prevalence ratio was relatively flat between 2005 and 2014, with less than 1% of the area achieving the 0.03 threshold; thereafter, areas with a female-incidence/male-prevalence ratio <0.03 increased to 8% in 2017. The incidence-mortality and incidence-prevalence ratios showed an emerging male microepidemic in the western part of the surveillance area (Figures 3 and 4).

## Discussion

South Africa is at the epicentre of the HIV pandemic, with the highest number of new infections and the most people living with AIDS.^4^ Over the past decade, the country has received substantial investment to scale up the world’s largest HIV treatment programme.^17^^,^^27^ Attention is now focused on interim progress to bring the HIV epidemic under control by 2030.^28^ One way to evaluate progress is to use single disease metrics that combine information on HIV-related mortality, HIV incidence and HIV prevalence. To this end, the UNAIDS has proposed the percentage reduction in incidence, the absolute incidence rate, the incidence-mortality ratio and the incidence-prevalence ratio, each having a specific target to mobilize efforts in the fight against HIV.^2^

Our results show impressive progress toward HIV epidemic control. However, epidemic transition targets were not achieved during the observation period. The 62% reduction in the number of new HIV infections among men (between 2012 and 2017) and the 34% reduction among women (between 2014 and 2017) are off track for the 75% target set by the UNAIDS for 2020. During these two time periods, respectively, we also observed significant declines in the absolute incidence rate: from 2.6 to 1.1 events per 100 person-years among men and from 4.9 to 3.1 events per 100 person-years among women. The decline in HIV incidence is consistent with the scale up of voluntary medical male circumcision (VMMC) and ART services in the surveillance area between 2009 and 2011.^6^ Nevertheless, the overall HIV incidence rate is currently well above the recommended target of 1 infection per 1000 person-years.

In line with the percentage and absolute incidence reductions, we also observed declines in the incidence-mortality ratio. The male incidence-mortality ratio peaked at 4.1 in 2013 before dropping to 3.1 in 2017 (a 24% reduction) and the female incidence-mortality ratio climbed to as high as 6.4 in 2013 before dropping to 4.3 in 2017 (a 33% reduction). Although these results indicate interim progress, the incidence-mortality ratios are high and above the threshold of 1. The failure to achieve epidemic control is due to a persistently high HIV incidence rate relative to a rapidly declining all-cause mortality rate among HIV-positive participants—a decline attributed to the survival benefits of ART.^29^^,^^30^ Because of the declining HIV-positive mortality, and therefore a steady increase in HIV prevalence, we observed reductions in the incidence-prevalence ratios over the observation period. Specifically, between 2012 and 2017, the male-incidence/female-prevalence ratio declined from 0.05 to 0.02. Compared with men, however, the female-incidence/male-prevalence ratio was markedly higher and fell from 0.24 to 0.13 during the same period. This result, when coupled with the higher HIV incidence, incidence-mortality ratio and HIV prevalence among women, confirms the disproportionate burden of HIV being experienced by women relative to men in sub-Saharan Africa.^6^^,^^14^^,^^31^

We observed substantial heterogeneity in the epidemic control metrics over place and time. Areas having an incidence-prevalence ratio <0.03 increased from 43% to 62% among men and from 1% to 8% among women between 2014 and 2017. Among men, an incidence-mortality ratio <1 was prevalent in 30% of the surveillance area in 2014, which increased to 47% in 2017. Both metrics also revealed an emerging male microepidemic in the west of the surveillance area, coinciding with the scale up of mining and other industrial activities.^32^ The epidemic was also concentrated in the south-east of the surveillance area, which borders a national highway and includes the KwaMsane township. Previous studies have reported high rates of HIV infection in this area.^33^ Using the epidemic metrics, our study highlights two hyperendemic areas that could benefit from targeted HIV prevention services.

Our study has several potential limitations. First, cohort study designs are recognized as the gold standard for incidence rate estimation,^34^ but it is often a challenge to maintain consistent participation rates over time.^35^ In the AHRI surveillance area, the cumulative testing rate was relatively high, with the percentage of participants with at least one HIV test increasing from 42% to 79% over the observation period. Importantly, 71% of HIV-negative participants who were eligible for entry in the incidence cohort contributed person-time to the current analysis. In a recent study, we rigorously show that reductions in HIV incidence were robust to participant selection, missed tests and drop-out.^6^ Second, it is possible that the large reductions in HIV incidence and HIV-related mortality could be explained by unobserved secular trends. However, population-based studies undertaken in Uganda, Kenya and South Africa have reported similar declines in HIV incidence following increased VMMC and ART coverage between 2011 and 2016.^5^^,^^7^^,^^8^ We have recently shown that the declines in the male and female HIV incidence rates in the surveillance area are consistent with the introduction of a local VMMC programme and opposite-sex ART coverage surpassing 35%.^6^ Given mounting evidence, the scale-up of HIV prevention services is likely the key driver of progress toward epidemic control in sub-Saharan Africa.^14^

The major strength of this study is our use of real-world data, collected from an entire population for over a decade, to estimate the four epidemic control metrics. This was made possible by AHRI’s comprehensive demographic surveillance system, in which fieldworkers have visited households, recorded deaths through verbal autopsies and tested participants for HIV on an annual basis since 2005. We leveraged these data to calculate key indicators such as the HIV prevalence, HIV incidence rate and the HIV-related mortality rate, which are needed for the incidence-mortality and incidence-prevalence ratios. Our empirically based results are an advance over recent work that has used mathematical models to derive estimates of the four metrics.^14^^,^^15^ As far as we know, no other study has presented, in the same analysis, empirical estimates of the four metrics and shown how they have varied over place and time.

In summary, there has been impressive progress toward HIV epidemic control in a hyperendemic South African setting. Nevertheless, expanded and optimized HIV prevention services are needed to reach key UNAIDS milestones. Our results, which show heterogeneity in the metrics over place and time, highlight areas that could benefit from these prevention services.

## Funding

This work was supported by two National Institute of Health (NIH) grants (R01HD084233 and R01AI124389). The Africa Health Research Institute’s Demographic Surveillance Information System and Population Intervention Programme is funded by the Wellcome Trust (201433/Z/16/Z), and the South Africa Population Research Infrastructure Network (funded by the South African Department of Science and Technology and hosted by the South African Medical Research Council). T.B. was supported by the Alexander von Humboldt Foundation through the endowed Alexander von Humboldt Professorship funded by the German Federal Ministry of Education and Research, as well as by the Wellcome Trust, the European Commission, the Clinton Health Access Initiative and the National Institutes of Health’s Fogarty International Center (D43-TW009775).

## Supplementary Material

dyz269_Supplementary_Data
