# Supplementary material for: The state of the HIV epidemic in rural KwaZulu-Natal, South Africa: a novel application of disease metrics to assess trajectories and highlight areas for intervention
Source: Int J Epidemiol. 2020 Jan 13;49(2):666–75. doi: 10.1093/ije/dyz269 (PMC7266544; doi:10.1093/ije/dyz269)
Supplement: dyz269_Supplementary_Data [file dyz269_supplementary_data.pdf]

Supplement Figures

Figure S1: Temporal trends in the HIV incidence rate (Panel A), all-cause HIV-positive mortality rate (Panel B), incidence-mortality ratio (Panel C), and HIV prevalence (Panel D) for all participants (aged 15–49 years) in the AHRI surveillance area (2005–2017). Panel E is the average of the male-incidence/female-prevalence and female-incidence/male-prevalence ratios shown in Figure 1.

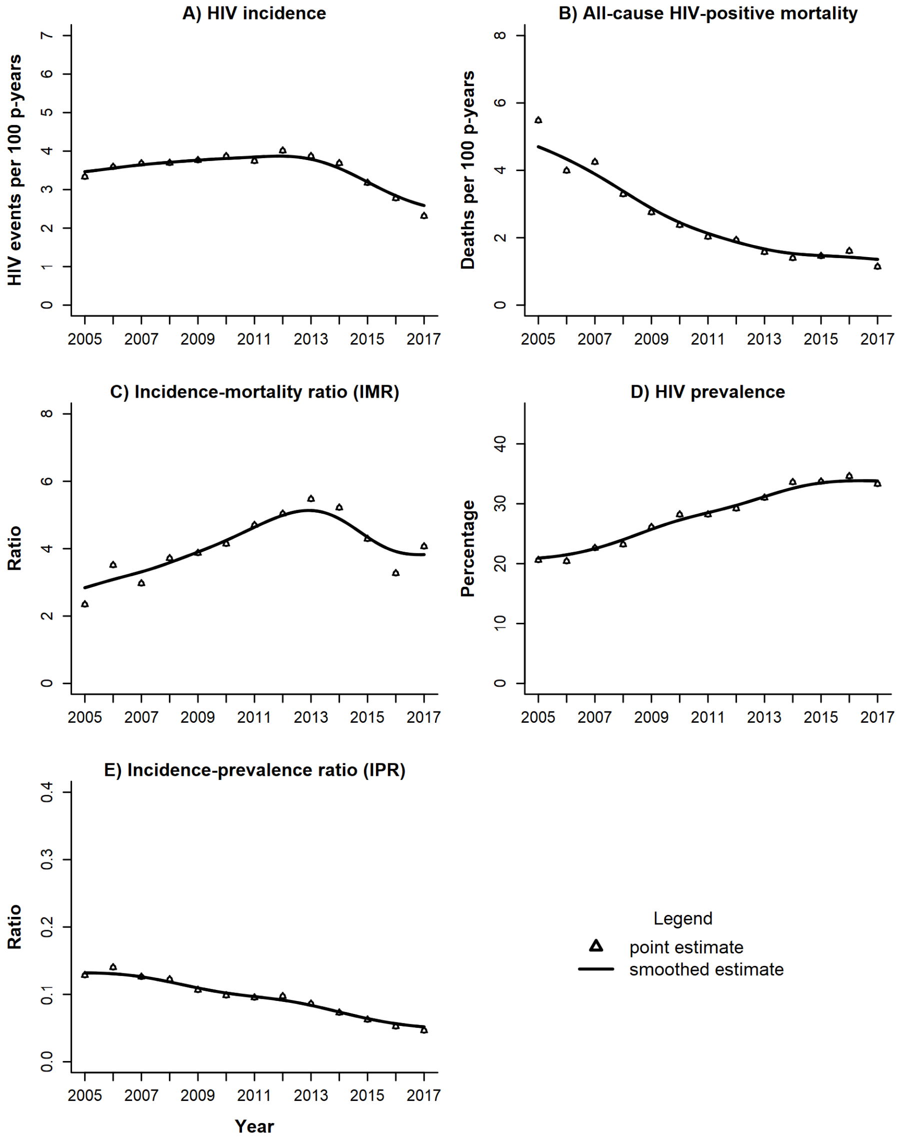

Figure S2: Shows the (same-sex) female-incidence/female-prevalence and male-incidence/male-prevalence ratios (IPR). Compared with the opposite-sex versions shown in Figure 1, the same-sex incidence-prevalence ratios are less informative about the disproportionate burden of HIV experienced by women relative to men in the study area.

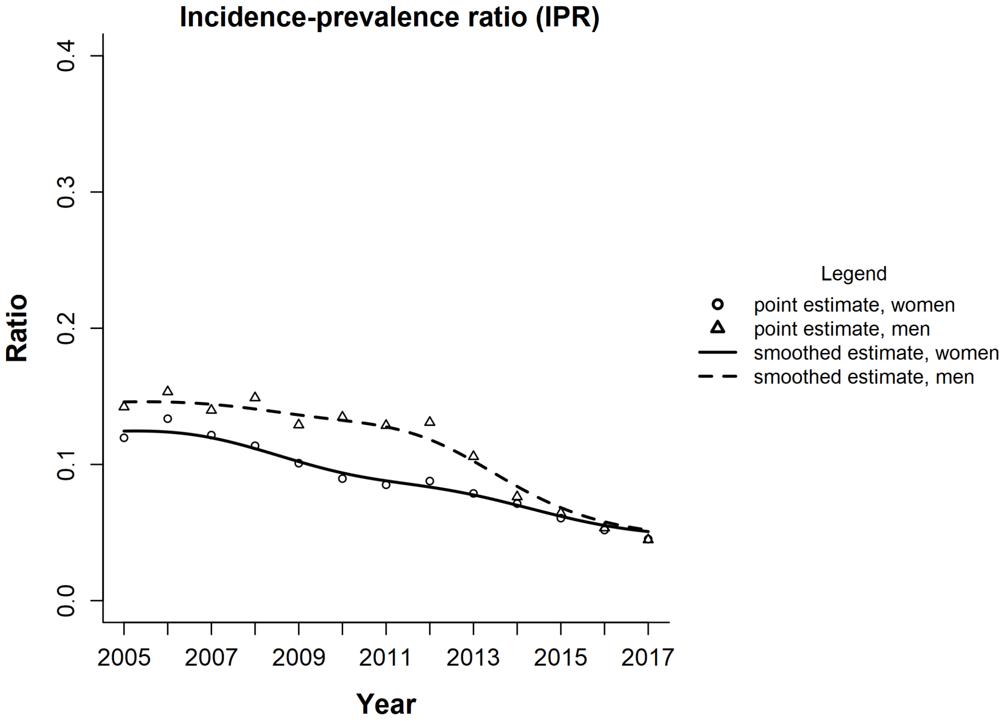

Supplement Tables

Table S1: Summary of the epidemic metrics for all participants (men and women) aged 15–49 years in the AHRI surveillance area (2005–2017).

	HIV Survey[†]					Incidence Cohort[§]			Mortality Cohort[¶]				
Year	N^T	HIV− Prev. (%)	HIV+ Prev. (%)	Exp. HIV− (N)	Exp. HIV+ (N)	HIV Inf. per 100 p-years	HIV Inc. Rate	Exp. HIV Inf. (N)	Deaths per 100 p-years	Mort. Rate	Exp. Death (N)	IMR[‖]	IPR[‡]
2005	63,799	79.4	20.6	50,656	13,142	254/7,614	3.33	1,688	170/3,102	5.48	720	2.34	0.178
2006	63,282	79.6	20.4	50,372	12,909	300/8,356	3.59	1,808	149/3,735	3.99	515	3.51	0.199
2007	63,155	77.4	22.6	48,881	14,273	313/8,490	3.68	1,799	174/4,097	4.25	606	2.97	0.182
2008	64,583	76.8	23.2	49,599	14,983	314/8,495	3.69	1,832	151/4,588	3.29	493	3.71	0.183
2009	66,423	73.9	26.1	49,086	17,336	304/8,079	3.76	1,847	136/4,940	2.75	477	3.87	0.153
2010	70,728	71.8	28.2	50,782	19,945	304/7,854	3.87	1,964	126/5,303	2.38	474	4.14	0.148
2011	70,306	71.8	28.2	50,479	19,826	285/7,601	3.74	1,889	121/5,967	2.03	402	4.70	0.141
2012	68,345	70.8	29.2	48,388	19,956	288/7,171	4.01	1,941	119/6,163	1.93	385	5.04	0.144
2013	70,577	69.0	31.0	48,698	21,878	280/7,221	3.87	1,884	103/6,547	1.57	344	5.47	0.126
2014	69,692	66.4	33.6	46,275	23,416	266/7,223	3.68	1,705	96/6,877	1.40	327	5.22	0.110
2015	73,643	66.3	33.7	48,825	24,817	224/7,054	3.17	1,550	107/7,350	1.46	361	4.29	0.103
2016	75,179	65.4	34.6	49,167	26,011	174/6,261	2.77	1,364	127/7,917	1.60	417	3.27	0.088
2017	71,012	66.7	33.3	47,365	23,646	113/4,884	2.31	1,095	93/8,163	1.14	269	4.06	0.075

[†] N^T gives the total number of participants that resided for >50% of the year in the surveillance area (irrespective of consent to HIV testing). HIV+ Prev. and HIV− Prev. represent the HIV-positive and HIV-negative prevalence, respectively. The expected number of HIV-negatives (column 5) is obtained by multiplying N^T (column 2) by the HIV-negative prevalence (column 3). The expected number of HIV-positives (column 6) is obtained by multiplying N^T (column 2) by the HIV-positive prevalence (column 4).

[§] Shows the number of observed HIV events and person-years of observation (column 7). The HIV incidence rate is per 100 person-years (column 8). The expected number of new HIV infections (column 9) is obtained by multiplying the expected number of HIV-negatives (column 5) by the HIV incidence rate/100 (column 8).

[¶] Shows the number of observed deaths among HIV-positive persons and the person-years of observation (column 10). The HIV-related mortality rate is per 100 person-years (column 11). The expected number of HIV-related deaths (column 12) is obtained by multiplying the expected number of HIV-positives (column 6) by the HIV-mortality rate/100 (column 11).

[‖] The incidence-mortality ratio (IMR, column 13) is obtained by dividing the expected number of new HIV infections (column 9) by the expected number of HIV-related deaths (column 12).

[‡] The incidence-prevalence ratio (IPR, column 14) is obtained by taking the average of the male-incidence/female-prevalence and female-incidence/male-prevalence ratios shown in column 14 of Table 2.

Supplement Methods

This section provides a more detailed overview of our methodology, with slightly different mathematical notation than the main text.

Let N^T denote the unique number of participants (irrespective of their HIV testing status) that were residents in the surveillance area for more than 50% of the year between 2005 and 2017. Also let $i = 1, \ldots, N$ denote the ith participant that consented to an HIV test, such that $N \leq N^T$. We calculate the HIV-positive prevalence as:

$$H_y^+ = \frac{\sum_{i=1}^{N} I(R_i)}{N_y}, \tag{1}$$

where R is the earliest HIV-positive test date, $I(R) = 1$ if R exists and occurs in year y otherwise $I(R) = 0$, and N_y is the number of participants that tested for HIV in year y. The annual HIV-negative prevalence is therefore $H_y^- = 1 - H_y^+$.

Next, we identified all participants with a first HIV-negative test followed by at least one HIV test result during the observation period. These repeat-testers comprise the HIV incidence cohort. Let U_{ik} denote the kth test date ($k = 1, \ldots, K$) for the ith repeat-tester ($i = 1 \ldots, n$) where $U_{i1} < U_{i2} < \cdots < U_{iK}$ and $K \geq 2$. Because of periodic testing, the seroconversion time T is unobserved and censored between the latest HIV-negative date (L) and the earliest HIV-positive date (R), where $L = max\{U_k : U_k < T\}$ and $R = min\{U_k : U_k \geq T\}$. Assuming a constant hazard of infection across the censoring interval $(L, R]$, we imputed a date T^* and right censored the data at either T^* or at L (for all unknown R). The justification for our imputation approach is provided elsewhere.[1,2] Let $\delta = 1$ denote that T^* occurs in year y ($y = 2005, \ldots, 2017$), otherwise $\delta = 0$, which we write as $I(\delta)$ where $I(\cdot)$ is an indicator function. Further, define Δ as the number of person-years since the earliest HIV-negative test date (U_1), calculated as either ($\Delta = T^* - U_1$) or ($\Delta = L - U_1$) divided by 365.25. We write the absolute incidence rate (IR) for year y as:

$$IR_y = \frac{\sum_{i=1}^{n} I(\delta_{ik})}{\sum_{i=1}^{n} \Delta_i}. \tag{2}$$

To account for the uncertainty of our imputation procedure, we generated $[j = 1, \ldots, 300]$ imputed datasets and took the average of the $IR_y^{[j]}$ estimates. We obtained standard errors and 95% confidence intervals for IR_y using Rubin's rules.[3] A well-cited target is to decrease the absolute incidence rate to less than one infection per 1,000 uninfected adults or person-years.[4,5]

We calculated the expected number of new infections by multiplying the absolute incidence rate with the expected number of HIV-negative participants in the population: $EI_y = \text{IR}_y \times (H_y^- \times N_y^T)$. We used this result to calculate the percentage change in the expected number of new infections over a given time-period:

$$EI\% = \frac{EI_{y_2} - EI_{y_1}}{EI_{y_1}} \times 100, \tag{3}$$

where the subscripts y_1 and y_2 denote a baseline year (time 1) and some future year (time 2), respectively. Targets for percentage reductions will vary by country and scale of the local epidemic. For example, under its 90-90-90 treatment targets, the UNAIDS aims to achieve a 75% reduction in the global number of new HIV infections between 2010 and 2020.[6]

We next calculated the AIDS-related mortality rate, which is needed for the incidence-mortality metric. Let V_{ik} denote the kth household visit date ($k = 1, \ldots, K$) for participants ($i = 1, \ldots, N$), where $V_{1k} < V_{2k} < \cdots < V_{iK}$. Denote the HIV-positive participant's date of death by D^+ and let $I(D^+)$ indicate that the death occurred in year y or not. We calculated the person-years of survival (ζ) from the earliest HIV-positive date (R) to death ($\zeta = D^+ - R$) or to the last household visit ($\zeta = V_K - R$) divided by 365.25. The AIDS-related mortality rate (MR) is:

$$MR_y = \frac{\sum_{i=1}^{N^+} I(D_{ik}^+)}{\sum_{i=1}^{N^+} \zeta_i}, \tag{4}$$

where N^+ is the number of participants that tested HIV-positive in year y, with $N^- + N^+ = N$. Let EP denote the expected number of HIV-positives, which we obtained by multiplying the total number of participants by the HIV-positive prevalence: $EP = N^T \times H^+$. We then obtained the annual expected number of deaths as the product of the AIDS-related mortality rate and the expected number of HIV-positives in the population: $ED_y = MR_y \times EP_y$. From the expected number of new infections and AIDS-related deaths, we calculate the annual incidence-mortality ratio as $IMR_y = \frac{EI_y}{ED_y}$. The threshold for epidemic control is an IMR <1, which is achieved when the number of new HIV infections (numerator) falls below the number of all-cause AIDS-related deaths (denominator) in a given year.[7]

For the incidence-prevalence ratio, we divided the expected number of new infections by the expected number of HIV-positives in the opposite-sex, such that $\text{IPR}_y = \frac{EI_y}{EP_y^o}$, where the o superscript denotes the opposite-sex (e.g., expected male infections divided by expected female HIV-positives). The threshold for epidemic control is an IPR <0.03. The value of 0.03 is arrived at by assuming that the average survival time of a newly infected person on ART is 33 years. To achieve epidemic control, fewer than one infection should occur over the 33 year-period, which translates into 1/33 or 3 new infections per 100 people living with HIV per year.[8]

Using the same methodology above, we computed geospatial versions of the IR_y, MR_y, H_y^+, and N_y^T. To do this, we used a moving two-dimensional Gaussian kernel of 3 km search radius,[9] the size of which was determined from previous work.[10] We identified the household coordinates of all participants and superimposed their HIV and mortality data on a geographic representation of the study area consisting of a grid of 1 km x 1 km pixels. Next, we calculated Gaussian weighted estimates of the IR_y, MR_y, H_y^+, and N_y^T and generated a raster grid for each. We computed H_y^-

by multiplying the raster grid of $1 - H_y^+$ with the raster grid of N_y^T. Similarly, we computed EI_y by multiplying the raster grids of IR_y, H_y^-, and N^T. We obtained ED_y by multiplying the raster grids of MR_y, H_y^+ and N_y^T. Lastly, we calculated the IMR_y for year y by dividing the raster grid generated for EI_y by the raster grid generated for ED_y, and used a similar procedure for IPR_y.

References

[1] Alain Vandormael, Adrian Dobra, Till Bärnighausen, Tulio de Oliveira, and Frank Tanser. Incidence rate estimation, periodic testing and the limitations of the mid-point imputation approach. *International Journal of Epidemiology*, 47(1):236–245, 2018.

[2] Alain Vandormael, Frank Tanser, Diego Cuadros, and Adrian Dobra. Estimating Trends in the Incidence Rate with Interval Censored Data and Time-dependent Covariates. *Statistical Methods in Medical Research: https://doi.org/10.1177/0962280219829892*, 2019.

[3] Joseph L Schafer. Multiple imputation: a primer. *Statistical Methods in Medical Research*, 8(1):3–15, 1999.

[4] Reuben M Granich, Charles F Gilks, Christopher Dye, Kevin M De Cock, and Brian G Williams. Universal voluntary HIV testing with immediate antiretroviral therapy as a strategy for elimination of HIV transmission: a mathematical model. *The Lancet*, 373(9657):48–57, jan 2009.

[5] UNAIDS. SDG indicator 3.3.1. *Sustainable Development Goals*, 2018.

[6] UNAIDS. *90-90-90: an ambitious treatment target to help end the AIDS epidemic.* UNAIDS, Geneva, 2014.

[7] PEPFAR. Strategy for Accelerating HIV/AIDS Epidemic Control (2017-2020). *President's Emergency Plan for AIDS Relief (PEPFAR)*, 2017.

[8] UNAIDS. *Making the end of AIDS real: Consensus building around what we mean by "Epidemic Control"*. Geneva: Joint United Nations Programme on HIV/AIDS, Geneva, 2017.

[9] LA Waller and CA Gotway. *Applied spatial statistics for public health data.* Wiley, Hoboken, NJ, 2004.

[10] F. Tanser, T. Barnighausen, G. S. Cooke, and M.-L. Newell. Localized spatial clustering of HIV infections in a widely disseminated rural South African epidemic. *International Journal of Epidemiology*, 38(4):1008–1016, aug 2009.
